# High Somatization Rates, Frequent Spontaneous Recovery, and a Lack of Organic Biomarkers in Post‐Covid‐19 Condition

**DOI:** 10.1002/brb3.70087

**Published:** 2024-10-08

**Authors:** Anna Tröscher, Patrick Gebetsroither, Marc Rindler, Vincent Böhm, Rainer Dormann, Tim von Oertzen, Anna Heidbreder, Raimund Helbok, Judith Wagner

**Affiliations:** ^1^ Department of Neurology Johannes Kepler University Linz, Kepler University Hospital Linz Austria; ^2^ Medical Directorate University Hospital Würzburg Würzburg Germany; ^3^ Department of Neurology Evangelisches Klinikum Gelsenkirchen, Academic Hospital University Essen‐Duisburg Gelsenkirchen Germany

**Keywords:** brain fog, EBV reactivation, post‐acute sequelae of COVID‐19 (PASC), post‐Covid

## Abstract

**Introduction:**

Many patients report neuropsychiatric symptoms after SARS‐CoV‐2 infection. Data on prevalence of post‐COVID‐19 condition (PCC) vary due to the lack of specific diagnostic criteria, the report of unspecific symptoms, and reliable biomarkers.

**Methods:**

PCC patients seen in a neurological outpatient department were followed for up to 18 months. Neurological examination, SARS‐CoV‐2 antibodies, Epstein–Barr virus antibodies, and cortisol levels as possible biomarkers, questionnaires to evaluate neuropsychiatric symptoms and somatization (Patient Health Questionnaires D [PHQ‐D]), cognition deficits (Montreal Cognitive Assessment [MoCA]), sleep disorders (ISS, Epworth Sleepiness Scale [ESS]), and fatigue (FSS) were included.

**Results:**

A total of 175 consecutive patients (78% females, median age 42 years) were seen between May 2021 and February 2023. Fatigue, subjective stress intolerance, and subjective cognitive deficits were the most common symptoms. Specific scores were positive for fatigue, insomnia, and sleepiness and were present in 95%, 62.1%, and 44.0%, respectively. Cognitive deficits were found in 2.3%. Signs of somatization were identified in 61%, who also had an average of two symptoms more than patients without somatization. Overall, 28% had a psychiatric disorder, including depression and anxiety. At the second visit (*n *= 92), fatigue (67.3%) and insomnia (45.5%) had decreased. At visit three (*n* = 43), symptom load had decreased in 76.8%; overall, 51.2% of patients were symptom‐free. Biomarker testing did not confirm an anti‐EBV response. SARS‐CoV‐2‐specific immune reactions increased over time, and cortisol levels were within the physiological range.

**Conclusion:**

Despite high initial symptom load, 76.8% improved over time. The prevalence of somatization and psychiatric disorders was high. Our data do not confirm the role of previously suggested biomarkers in PCC patients.

## Graphical abstract

Numerous patients report neuropsychiatric symptoms following SARS‐CoV‐2 infection, with fatigue, stress intolerance, and cognitive deficits being predominant. In a cohort of 175 patients, 61% exhibited somatization, frequently accompanied by higher symptom load. Questionnaire assessments and self‐reported symptom load showed a reduction in symptoms over time for the majority of patients. Biomarker testing did not support Epstein–Barr virus involvement, and cortisol levels remained within the normal range.

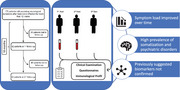

## 1 Introduction

A substantial number of patients report persistent or new‐onset symptoms months after SARS‐CoV‐2 infection, which are defined as post‐Covid‐19 condition (PCC) (Nalbandian et al. 2021; Huang et al. 2021; Altmann et al. 2023). Fatigue, headache, cognitive complaints, as well as depression and anxiety are the most common manifestations of PCC and are reported months or even years after COVID‐19 (Altmann et al. 2023; Taquet et al. 2021; Giussani et al. 2024). In addition, anosmia or ageusia may persist for months, which distinguishes PCC from chronic fatigue syndrome, where olfactory symptoms do not occur (Nalbandian et al. 2021; Huang et al. 2021; Komaroff and Lipkin 2023). Many studies have investigated PCC outcome after several months (Taquet et al. 2021; Giussani et al. 2024; Wahlgren et al. 2023; Kim et al. 2023); however, more recent studies have also investigated long‐term outcome of PCC patients over 2 years (Wahlgren et al. 2023; Kim et al. 2023). In these studies, most common symptoms at 24 months are still similar to the early follow‐ups. Although persisting symptoms seem particularly frequent after severe disease, a considerable amount of patients suffering from PCC are middle‐aged, non‐hospitalized, with a mild disease course without pre‐existing comorbidities (Graham et al. 2021; Blomberg et al. 2021). Furthermore, depending on the viral strain and the study design, the incidence of PCC varies substantially, with rates being highest after the first waves (up to 65%) and lowest with the latter strains (Kahlert et al. 2023; Du et al. 2022). Incidence rates largely differ depending on the investigated population and the specificity of the definition used, ranging between 7.5% and 41% (Nittas et al. 2022). Another study found only 2.9% prevalence of self‐reported PCC (West et al. 2020; Høeg, Ladhani, and Prasad 2023). A UK‐based team found a 5% prevalence of self‐reported PCC symptoms. However, also 3.4% of seronegative patients reported PCC symptoms, suggesting an overestimation of PCC (Høeg, Ladhani, and Prasad 2023; ONS 2021). In a PCC outcome clinic during the early phase of the pandemic, only anosmia was significantly more common in SARS‐CoV‐2 seropositive patients, whereas other neuropsychiatric symptoms were equally distributed in seronegative and seropositive patients (Graham et al. 2021). Hence, somatic symptom disorder (SSD), the excessive focus, and maladaptive thoughts regarding experienced symptoms in PCC, has recently gained interest and could be driven by anxiety and enhanced introspection of the population (Kachaner et al. 2022; Horn et al. 2023; Ludwig et al. 2023). Several studies have investigated the percentage of PCC patients affected by a somatization disorder and found 10%–65% of PCC patients positive on somatization evaluation questionnaires, such as the Patient Health Questionnaire‐15 or the Statistical Manual of Mental Disorders‐5 (Kachaner et al. 2022; Horn et al. 2023; Fleischer et al. 2022).

Pathomechanisms of PCC are complex and still incompletely understood. Hypotheses include autoimmunity, microvascular thrombi, reactivation of latent CNS viruses, such as Epstein–Barr virus (EBV), or a lower immune response against SARS‐CoV‐2 (Gold et al. 2021; Monje and Iwasaki 2022; Spatola et al. 2023). However, none of the theories are robust and support high specificity in the diagnosis of PCC, whereas increasingly more evidence is being accumulated in favor of an SSD in a large proportion of patients (Høeg, Ladhani, and Prasad 2023; Fleischer et al. 2022; Sykes et al. 2021). Therefore, most clinicians still rely on the clinical judgment and exclusion of other diseases instead of robust diagnostic criteria.

To address these issues, we prospectively studied a cohort of PCC patients and used standardized questionnaires to assess fatigue, insomnia, daytime sleepiness, cognition, and psychosocial factors longitudinally. Repeated blood examinations for routine lab parameters and antibody titer measurements were performed to identify PCC‐specific biomarkers. We evaluated the frequency of the most common neuropsychiatric symptoms and if somatization, based on the Patient Health Questionnaires D (PHQ‐D) questionnaire, contributes to symptom load and recovery. Furthermore, we hypothesize that current postulated biomarkers based on EBV reactivation and the immune response against SARS‐CoV‐2 are not specific enough for a clear diagnosis of PCC.

## 2 Materials and Methods

### 2.1 Patient Cohort

Between May 2021 and February 2023, 175 consecutive adult patients (≥ 12 weeks after diagnosis of SARS‐CoV‐2 infection) were recruited (100% of patients visiting the clinic) in a monocentric study in the PCC outcome clinic for neurological PCC symptoms at the Department of Neurology of the Johannes Kepler University, Kepler University Hospital Linz, Austria. Patients came to the clinic upon referral of a general practitioner or other specialist and had generally undergone various clinical assessments and diagnostic tests prior to the visit in our clinic. The referral criteria were consistent with the WHO definition of PCC, requiring an acute SARS‐CoV‐2 infection that occurred at least 12 weeks prior and ongoing symptoms without apparent alternative explanation (World Health Organization 2022). Furthermore, neuropsychiatric symptoms had to be the dominant complaint. No further restrictions applied. All patients had confirmed SARS‐CoV‐2 infection. Patients were not included if they had pre‐existing myalgic encephalomyelitis/chronic fatigue syndrome or other severe pre‐existing co‐morbidities, such as an autoimmune disease requiring constant immune suppression. Sociodemographic data were recorded in all patients. Patients were split into mild/moderate disease course (home‐isolated) or severe disease course (hospitalized, ICU). Patients had a median of one (range: 1–6 visits) visit, of which only the first, second, and third visits were included in this study due to high drop‐out rates. The first visit was 9.87 (median; IQR = 6.95–13.28) months after the acute infection, and the second one was 3.25 (median; IQR = 1.85–5.01) months later. The last visit was 17.57 (median; IQR = 12.87–22.05) months after the acute infection (11.16 months after the first visit, IQR = 7.93–13.41). Concerning physical therapy, patients were informed about the concept of pacing and, if required, referred to specialized PCC rehabilitation centers. Furthermore, patients were referred to a standardized PCC patient education group led by neuropsychologists of the Department of Neurology, Kepler University Hospital Linz, Austria, and to the outpatient clinic for somatization disorders of the Department of Psychiatry, Kepler University Hospital Linz, Austria.

### 2.2 Ethics Statement

This study was approved by the Ethics Committee of the Medical Faculty, Johannes‐Kepler University, Linz, Austria (1161/2021). This study confirms the World Medical Organization Declaration of Helsinki.

### 2.3 Clinical Examination

All patients underwent a standardized neurological examination by a neurologist. 127/175 (73%) patients underwent standard laboratory diagnostics, including total blood count, differential blood count, iron metabolism, liver and kidney function parameters, coagulation profile, vitamin B12, folic acid, C‐reactive protein, serum electrolytes, thyreotropin, serum glucose, serum cortisol, serum creatinin kinase, and lactate dehydrogenase. Blood sampling mean time was 10:12 a.m. (IQR = 9:00–11:24 a.m.). Further diagnostic procedures (cerebral imaging [CT or MRI], electroneurography, electromyography, EEG, and CSF examination) were performed as needed.

### 2.4 Symptoms

Clinical neurological examination was performed during Visit 1 and re‐performed at every follow‐up visit depending on the complaints. Subjective symptoms were recorded during clinical examination. The most common symptoms (≥ 10 patients affected at first visit) were categorized into 13 groups: fatigue, subjective stress intolerance (sStIn), daytime sleepiness, insomnia, cognition deficits, pain (including headache and myalgias), chest oppression, dyspnea, hyposmia, parosmia, tachycardiac episode, vertigo, and tinnitus.

### 2.5 Questionnaires

Questionnaires regarding sleep/fatigue and sleepiness were administered: The “Fatigue Severity Score” (FSS, cut‐off: 36/63 points) (Krupp et al. 1989), “Insomnia Severity Index” (ISI; subthreshold insomnia: 8–14/28 points, moderate clinical insomnia: 15–21/28 points, severe clinical insomnia: 22–28 points) (Morin et al. 2011), and the “Epworth Sleepiness Scale” (ESS, cut‐off: 10/24 points) (Johns 1991) were used to determine fatigue, insomnia, and daytime sleepiness and were repeated at the first follow‐up. For assessment of memory and cognition, the “Montreal Cognitive Assessment” (MoCA) was performed. The PHQ‐D was used to evaluate a broad variety of psychosocial and psychological factors at the first visit. It is well validated in patients with psychiatric and non‐psychiatric diseases, in the clinic as well as in research (Fleischer et al. 2022; Gräfe et al. 2004; Spitzer, Kroenke, and Williams 1999). This test evaluates seven different domains of psychiatric disorders: 1. somatization (sensitivity: 77%, specificity: 83%), 2. depression (sensitivity: 75%, specificity: 90%), 3. anxiety (sensitivity: 67%, specificity: 94%), 4. panic disorders (sensitivity: 73%, specificity: 98%), 5. other anxiety/fear states (sensitivity: 89%, specificity: 82%), 6. eating disorders (sensitivity: 58%, specificity: 94%), and 7. alcohol abuse (sensitivity: 57%, specificity: 96%) (Gräfe et al. 2004).

### 2.6 Impact of Somatization on Subjective Symptoms

According to the ICD‐10 catalog, somatization disorders are defined as the repeated presentation of physical symptoms together with persistent requests for medical investigations, in spite of repeated negative findings and reassurances by doctors that the symptoms have no physical basis. If any physical disorders are present, they do not explain the nature and extent of the symptoms or the distress and preoccupation of the patient. This discrepancy as well as the accompanying affective and cognitive components were explicitly elicited during the clinical consultation (ICD 2019). To evaluate the impact of somatization on the symptom load, we calculated the frequency distribution of all recorded symptoms according to somatization status. Furthermore, we evaluated the overall complaint load depending on the somatization status.

### 2.7 Antibody Titer Measurements

Serum EBV nuclear‐1 antigen immunoglobulin G (EBNA‐1 IgG) as well as the SARS‐CoV‐2 IgG against the nucleocapsid protein were measured with a chemiluminescent microparticle immunoassay (CMIA) using the Abbott Architect System EBV EBNA‐1 IgG detection kit (Abbott Laboratories, Ref. Nr.: 3P67) and the SARS‐CoV‐2 detection kit (Abbott Laboratories, Ref. Nr.: 6R86‐22) according to the manufacturer's instructions. EBNA‐1 IgG is given as the ratio of the relative luminescence intensity of the sample over the control (S/CO). The SARS‐CoV‐2 IgG is shown as an arbitrary unit (AU) per mL.

### 2.8 Statistics

Statistics were done with GraphPad Prism (version 6.1). Continuous variables were tested for normality with the D'Agostino and Pearson omnibus normality tests. Friedmann test with multiple comparisons and Dunn's post hoc correction were performed to evaluate the complaint load over time. A Wilcoxon‐signed rank test was used to evaluate if score results were significantly higher/lower than the pathological cut‐off value (36 for FSS, 15 for ISI, and 10 for ESS). For longitudinal analysis of score results, the Wilcoxon matched‐pair signed rank test was used. Differences between hospitalized and non‐hospitalized patients were tested with the Mann–Whitney *U*‐test. Fisher's exact test was used to test if symptom frequency differed between somatization positive or negative patients at both timepoints. To evaluate if there is a difference in the number of symptoms (complaint load) between somatization positive and negative, a two‐sided *t*‐test was performed. Mediator analyses were done with the DATAtab online statistics calculator (DATAtab e.U. Graz, Austria) (Team Data 2024). To evaluate the effect of various psychiatric diseases on the development of symptoms, we combined all positive results for “depression,” “anxiety,” and “other fear states,” as well as previously diagnosed psychiatric diseases. All non‐parametric data are shown as median ± IQR. Parametric data are shown as mean ± SD or min/max for boxplots. Symptom frequencies are indicated as percentages of patients affected and are therefore shown as a single value without error bars. This patient cohort represents a convenience sample. No formal sample size calculation was performed prior to the study initiation.

## 3 Results

### 3.1 Demographic Data

**FIGURE 1:**
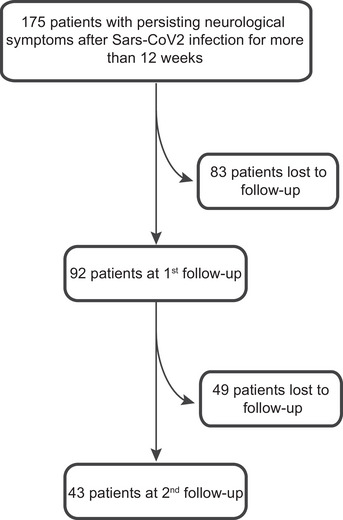
Flow chart of patient numbers included in the study and drop‐outs among follow‐ups.

A total of 175 consecutive patients with a median age of 42 years (IQR = 33–52) were included in this study, of which 78% (*n *= 136) were female. The majority had mild‐to‐moderate COVID‐19 infection, and only 15/175 (9%) were hospitalized or received ICU treatment (4/175, 2%). Median time between COVID‐19 infection and the first visit was 9.87 (IQR = 6.95–13.28) months. Although invited, only 92/175 (53%) and 43/175 (25%) had at least one follow‐up visit after 3.25 (median; IQR = 1.85–5.01) months and another 11.16 months after the first visit (IQR = 7.93–13.41), respectively (Figure 1).

Overall, 97% of the patients had had SARS‐CoV‐2 infection confirmed by RT‐PCR or lateral‐flow antigen tests; the remaining 3% were tested positive for SARS‐CoV‐2 antibodies. Before the SARS‐CoV‐2 infection, 108/112 (96%) of the patients were employed or studying at university. Of those, 75 (69%) had not been able to return to work/university in full capacity at first presentation due to residual symptoms (Table 1).

**TABLE 1:** Demographic data of post‐COVID‐19 condition (PCC) patients.

Demographics	
Sex (m/w) [w %]	39/136 [77.71%]
Age (years; mean ± SD)	42.54 ± 12.04
Vaccinated (yes/no/unknown) [%]	109/20/46 [62.29/11.43%/26.28%]
Hospitalized (yes/no) [yes %]	15/160 [8.57%]
ICU (yes/no) [yes %]	4/171 [2.29%]
Employed/Studying before infection (yes/no/unknown) [%]	108/4/63 [61.71/2.29/36.00%]
Unemployed/Reduction of working hours due to PACS at first visit (yes/no) [yes %] (*n *= 108)	75/33 [69.44%]
**General neuropsychological assessment**	
Focal neurological deficit (yes/no) [yes %]	5/170 [2.86%]
Psychiatric diagnosis (yes/no) [yes %]	48/127 [27.43%]
Cerebral Imaging (yes/no) [yes %]	60/175 [34.29%]
Other diagnosis made possibly explaining symptoms (yes/no) [yes %]	5/168 [2.86%]

### 3.2 Clinical Examination and Laboratory Results

**FIGURE 2:**
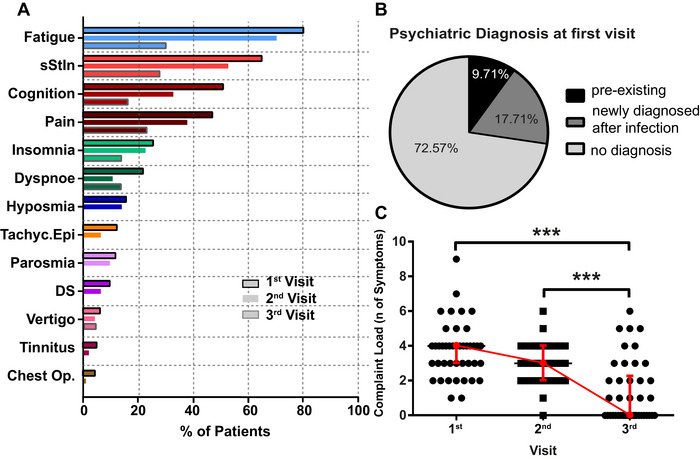
Longitudinal evaluation of symptoms: The 13 most frequent self‐reported symptoms over 3 follow‐ups are shown, with the first visit indicated by black border, the second visit shown without a border color, and the third visit indicated by gray border. (A) Psychiatric diagnosis at the first visit; (B) complaint load analyzed over three visits in paired analysis (*n *= 43; data shown as median ± IQR; Friedman test *F* = 31.84; *p *< 0.0001; Dunn's multiple comparison correction: first vs. second visit: ns; first vs. third visit *p *< 0.0001; second vs. third visit *p *< 0.001) (C). Chest Op. = chest oppression (first visit *n *= 175, second visit *n *= 92, third visit *n *= 43), DS = daytime sleepiness. sStIn = subjective stress intolerance, Tachy.Epi = tachycardiac episodes.

At the first visit, 3% of patients reported unspecific neurological symptoms like weakness or hypoesthesia (one patient had intermitting trigeminal hypoesthesia, one patient had hypoesthesia and hypopallesthesia in the lower extremities, one had paraesthesia of the upper extremities, one had dysesthesia of the upper and lower extremities, and one had hypoesthesia of the right side of the face, Table 1). In 48/175 (28%) of patients, a psychiatric disorder was present, of which 28/175 (18%) were newly diagnosed after the infection (Table 1, Figure 2B). In 60/175 (34%) patients, cerebral imaging was performed (e.g., dementia assessment), but none showed pathological findings (Table 1). In 3% of patients, a diagnosis possibly explaining the symptoms ascribed to PCC was made during the assessments: in three patients, small fiber neuropathy was diagnosed (explaining pain and tingly sensations in hands/feet), one patient had an ataxic gait disorder (explaining instability when walking), and one patient had hypothyroidism (explaining fatigue). Serological findings were made in two patients (1%; anticardiolipin antibodies, increased Lupus anticoagulant, and GAD65‐antibodies). One patient reporting chest pain had a scintigraphy indicating a corpus sterni contusion. In the routine blood analyses, no specific changes were detected (Table S1).

### 3.3 Symptoms and Neuropsychiatric Diagnoses at the First Visit

The most common self‐reported symptom was fatigue (88%), followed by subjective cognitive deficits (78%), and sStIn (74%). Less commonly reported symptoms included pain, insomnia, and hyposmia (Table 2, Figure 2A).

**TABLE 2:** Symptom frequency in all three visits.

	First visit (*n *= 175)	Second visit (*n *= 92)	Third visit (*n *= 43)
Fatigue (yes/no) [yes %]	140/175 (80.0%)	65/92 (70.7%)	13/43 (30.2%)
Subj. Stress Intolerance (yes/no) [yes %]	115/175 (65.7%)	52/92 (56.5%)	12/43 (27.9%)
Cognition (yes/no) [yes %]	89/175 (50.9%)	30/92 (33.0%)	7/43 (16.3%)
Pain (yes/no) [yes %]	82/175 (46.9%)	35/92 (38.0%)	10/43 (23.3%)
Insomnia (yes/no) [yes %]	44/175 (25.1%)	21/92 (22.8%)	6/43 (14.0%)
Dyspnoea (yes/no) [yes %]	38/175 (21.7%)	10/92 (10.9%	6/43 (14.0%)
Hyposmia (yes/no) [yes %]	27/175 (15.4%)	13/92 (14.1%)	0/43 (0.0%)
Parosmia (yes/no) [yes %]	20/175 (11.4%)	9/92 (9.8%)	0/43 (0.0%)
Tachycardiac Episodes (yes/no) [yes %]	22/175 (12.6%)	6/92 (6.5%)	0/43 (0.0%)
Daytime Sleepiness (yes/no) [yes %]	18/175 (10.3%)	6/92 (6.5%)	0/43 (0.0%)
Vertigo (yes/no) [yes %]	10/175 (5.7%)	4/92 (4.4%)	2/43 (4.7%)
Tinnitus (yes/no) [yes %]	10/175 (5.7%)	2/92 (2.2%)	0/43 (0.0%)
Chest oppression (yes/no) [yes %]	8/175 (4.6%)	1/92 (1.1%)	0/43 (0.0%)

**FIGURE 3:**
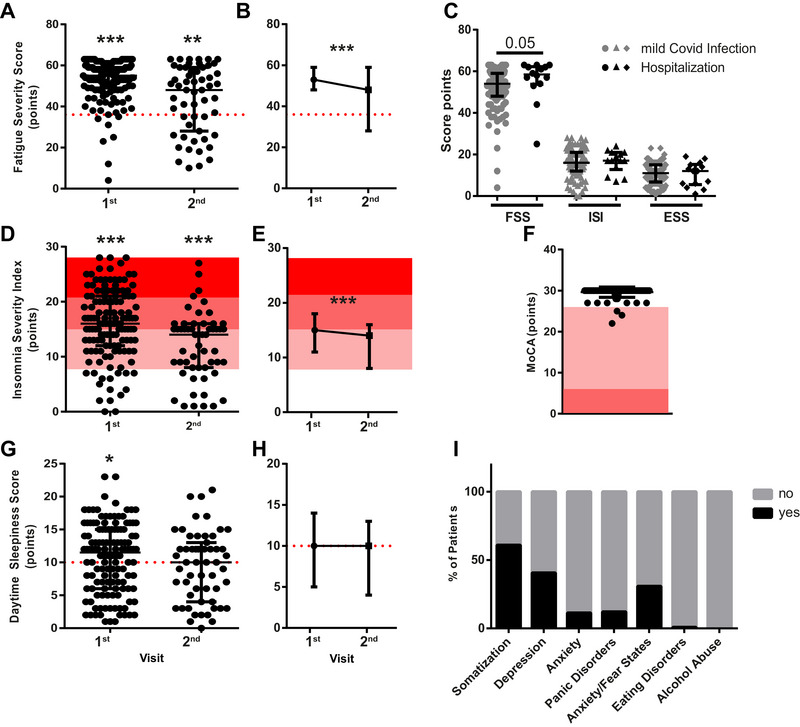
Longitudinal evaluation of scores: FSS was significantly higher than the pathological cut‐off (indicated by the red dotted line; data shown as median ± IQR; Wilcoxon‐signed rank test first visit *p* < 0.0001 and second visit *p *= 0.0014). (A) There is a significant decrease in FSS points achieved over time (data shown as median ± IQR; Wilcoxon matched‐pairs signed rank test *p *< 0.001). (B) There is no difference between hospitalized and mildly/moderately affected patients (data shown as median ± IQR; Mann–Whitney test FSS *p *= 0.0506; ISI *p *= 0.589; ESS *p* = 0.98). (C) ISI was significantly higher than the pathological cut‐off (threshold insomnia indicated in the light red area, moderate clinic insomnia indicated in the medium red, and severe clinical insomnia indicated in the dark red area; median ± IQR; Wilcoxon‐signed rank test first visit *p *< 0.0001, second visit *p *< 0.0001). (D) There is a significant decrease in ISI points achieved over time (data shown as median ± IQR; Wilcoxon matched‐pairs signed rank test *p *< 0.0001). (E) Median MoCa scores were not lower than the physiological cut‐off (light red area indicating cognitive impairment, medium red area indicating severe cognitive impairment; data shown as median ± IQR). (F) ESS was significantly elevated compared to the physiological cut‐off in the first visit (indicated by the red‐dotted line; data shown as median ± IQR; Wilcoxon‐signed rank test first visit *p* = 0.04; second visit *p* = 0.12). (G) There is no change in ESS points achieved over time (data shown as median ± IQR; Wilcoxon matched‐pairs signed rank test *p* = 0.3). (H) Patient Health Questionnaires D (PHQ‐D) questionnaire indicating percentage of patients affected by Item (for all graphs: first visit *n* = 132, second visit *n* = 55, in matched analysis *n* = 55). (I) % of patients who scored positive/negative in the single items of the PHQ‐D questionnaire. ESS, Epworth Sleepiness Scale.

The symptom load was determined by questionnaires, which were filled out by 132/175 (75%) patients at the first visit. In 125/132 (95%), fatigue was present, and FSS scores were significantly elevated compared to the pathological cut‐off (median = 55, IQR = 49–59; *p *< 0.0001, Figure 3A). Severe clinical insomnia was diagnosed in 29/132 (22.0%) patients, moderate insomnia in 53/132 (40.2%), subthreshold insomnia in 35/132 (26.5%) patients, and 15/132 (11.4%) patients were not affected by insomnia. Median ISI scores were significantly elevated compared to the pathological cut‐off (median = 16, IQR = 12–21; *p *< 0.0001; Figure 3D). Daytime sleepiness was diagnosed in 58/132 (44.0%) patients, and median scores were significantly elevated to the pathological cut‐off (median = 11.5, IQR = 6–15; *p *= 0.04, Figure 3G). When comparing hospitalized patients with mildly affected patients, no significant difference was found in either FSS, ISI, or ESS scores (Figure 3C). Cognitive impairment evaluated by MoCA found impairment in 3/132 (2.3%) of patients, who had no previous diagnosis of dementia, were of average age and had only mild/moderate disease course but suffered from severe subjective cognitive deficits (median = 30, IQR = 30–30, Figure 3F). The PHQ‐D questionnaire revealed that 75/123 (61%) patients were positive for somatization, 50/123 (40.7%) for depression, 15/123 (12.2%) for anxiety, 14/123 (11.4%) for panic attacks, 38/123 (30.9%) for other anxiety/fear states, and 1/123 (0.8%) for eating disorder and non‐alcohol abuse (Figure 3I).

### 3.4 Symptom Load at the Second Visit

Only 92/175 (53%) patients had follow‐up visits. The most common self‐reported symptom was fatigue (70.7%). sStIn still affected 56.5%, and a third (33.0%) reported subjective cognitive deficits (Table 2, Figure 2A).

The questionnaires were filled out in 55/92 (60.0%) of the patients at the second visit. Of those, 37/55 (67.3%) exceeded the cut‐off according to the FSS, and median levels were significantly elevated compared to the pathological cut‐off (median = 48, IQR = 29.5–58; *p* = 0.0014, Figure 3A). When comparing the FSS scores of both visits of the 55 patients who filled out the questionnaire twice, a significant reduction in FSS severity was observed (*p* = 0.0004; Figure 3B).

Severe clinical insomnia affected 3/55 (5.5%) of patients at the second visit, 22/55 (40.0%) had moderate and 18/55 (32.7%) had subthreshold insomnia according to the ISI. Median values were significantly elevated compared to the pathological cut‐off (median = 14, IQR = 8–15.5; *p* < 0.0001; Figure 3D). When comparing the ISI scores of both visits of the 55 patients who filled out the questionnaire twice, a significant reduction in ISI severity can be observed (*p *= 0.0003; Figure 3E).

The ESS revealed no significant increase of daytime sleepiness at the second visit, although 30/55 (54.6%) had scores > 10/24 (median = 10, IQR 4.5–12.5; *p *= 0.12, Figure 3G). When comparing the ESS scores of both visits of the 55 patients who filled out the questionnaire twice, no significant difference between the first and second visits can be observed (*p *= 0.3; Figures 3H).

### 3.5 Symptoms at the Third Visit

Out of 175, 43 (25.0%) had a second follow‐up. Fatigue and sStIn were still the most common symptoms but only present in one‐third of the patients. Other symptoms were reported much less frequently or were no longer present (Table 2, Figure 2A). For an overview of the subjective symptom load over time, the median complaint load was calculated. At the first visit, a median of four symptoms were reported (range 1–9). At the second visit, the complaint load had decreased to three (range 0–6), and on the third visit, it significantly dropped to zero (range 0–6, *p *< 0.0001; multiple comparison correction: first vs. second visit: ns; first vs. third visit *p *< 0.0001; second vs. third visit *p *< 0.001; Figure 2C).

Due to the high drop‐out rate (only 21/175 [12%] patients filled out the questionnaires three times), no score analyses were performed for the third visit.

### 3.6 Somatization Increases Symptom Frequency

**FIGURE 4:**
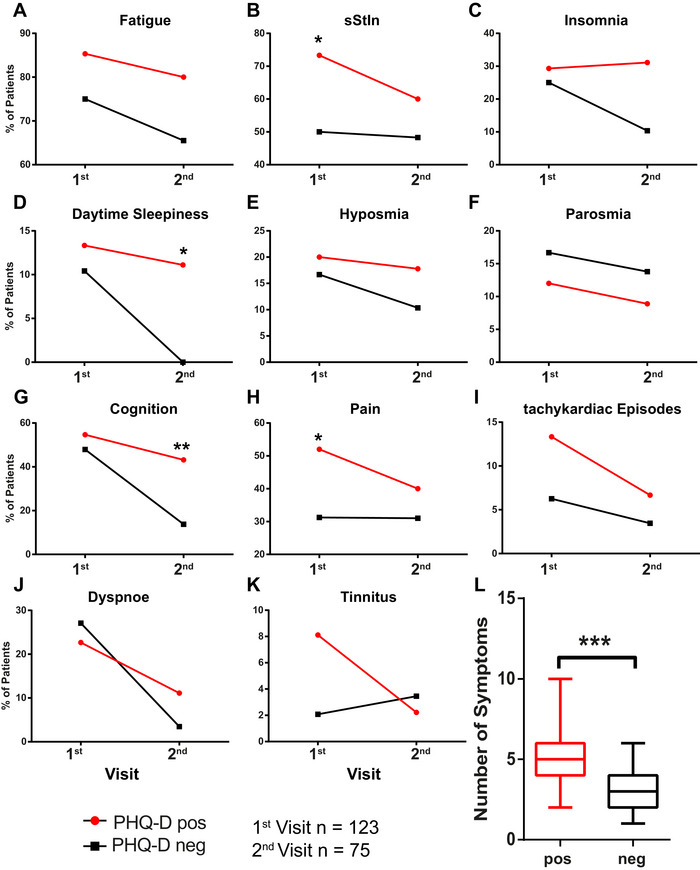
(A–K) Subjectively reported symptoms in PHQ‐D positive and negative patient cohort: all values are indicated as percentage of patients affected over time (first visit *n *= 123 (PHQ‐D negative *n *= 40, PHQ‐D positive *n *= 75), second visit *n* = 75 (PHQ‐D negative *n *= 29, PHQ‐D positive *n* = 45), Fisher's Exact Test, *p < 0.05, **p < 0.01). (L) Complaint load is significantly higher in PHQ‐D item 1 positive patients than in negative patients (data shown as mean, min–max; *t*‐test *p *< 0.0001). PHQ‐D, Patient Health Questionnaires D.

To evaluate the effect of somatization on the prevalence of PCC symptoms, we split the patient cohort according to the result on the PHQ‐D somatization questionnaire (positive or negative). We evaluated the longitudinal progress of symptoms in the two cohorts. sStIn and pain were significantly higher in the somatization‐positive cohort at the first visit (Figure 4B,H, Table 3). At the second visit, daytime sleepiness and cognition affected the somatization‐positive cohort significantly more (Figure 4D,G, Table 3). The overall complaint load at the first visit was significantly higher in the somatization‐positive cohort than in the negative patients (Figure 4L).

**TABLE 3:** Somatization positive and somatization negative patients have different symptom frequency and longitudinal change in most common post‐COVID‐19 condition (PCC) symptoms.

	First visit		Second visit	
[yes/no (yes %)]	Somatization negative	Somatization positive	Somatization negative	Somatization positive
Fatigue	36/12 (75%)	64/11 (85.33%)	19/10 (65.52%)	36/9 (80%)
sStIn	24/24 (50%)	55/20 (73.33%)	15/15 (48.28%)	27/18 (60%)
Insomnia	12/36 (25%)	22/53 (29.33%)	3/26 (10.34%)	14/31 (31.11%)
Daytime Sleepiness	5/43 (10.42%)	10/65 (13.33%)	0/29 (0%)	5/40 (11.11%)
Hyposmia	8/40 (80%)	15/60 (20%)	3/26 (10.34%)	8/37 (17.78%)
Parosmia	8/40 (16.67%)	9/66 (12%)	4/25 (13.79%)	4/41 (8.89%)
Cognition	23/25 (47.92%)	41/34 (54.67%)	4/25 (13.79%)	19/25 (43.18%)
Pain	15/33 (31.25%)	39/36 (52%)	9/20 (31.03%)	18/27 (40%)
Tachycardiac Episodes	3/45 (6.25%)	10/65 (13.33%)	1/28 (3.45%)	3/42 (6.67%)
Dyspnoe	13/35 (27.08%)	17/58 (22.67%)	1/28 (3.45%)	5/40 (11.11%)
Tinnitus	1/47 (2.08%)	6/68 (8.11%)	1 (28 (3.45%)	1/44 (2.22%)

Abbreviation: sStIn: subjective stress intolerance.

As psychiatric diseases and somatization are often interlinked, we evaluated if not only a positive test result on the somatization part of the PHQ‐D questionnaire affected the number of symptoms, but if psychiatric diseases act as a modulating factor. To this end, we performed a mediator analysis (Table 4). We could show that although the total effect of somatization on the number of symptoms was statistically significant (*c*: effect 0.61, *p *< 0.05), no modulating effect of psychiatric comorbidities was found (*c′*: effect 0.79, *p *= 0.1; *a* and *b*: effect − 0.18, *p *= 1.0).

**TABLE 4:** Results of mediator analysis of somatization on number of symptoms, modulated by psychiatric comorbidities, gender, and age.

		Effect	*p* value
**Total effect of somatization on number of symptoms (*c*)**		0.61	0.04*
**Psychiatric comorbiditie**s	Direct effect on number of symptoms (*c′*)	0.79	0.102
	Indirect effect on number of symptoms (*a* and *b*)	− 0.18	1
**Gender**	Direct effect on number of symptoms (*c′*)	0.63	0.037*
	Indirect effect on number of symptoms (*a* and *b*)	− 0.01	1
**Age**	Direct effect on number of symptoms (*c′*)	0.59	0.047*
	Indirect effect on number of symptoms (*a* and *b*)	0.03	0.647

Additionally, we investigated if sociodemographic differences modulate the number of symptoms developed. We repeated the mediator analysis for gender and found that the direct effect (*c′*) was indeed affected by the gender as the effect increased slightly (effect 0.63, *p *< 0.05). The indirect effect on the number of symptoms was not significantly altered (Table 4). When using the age as a moderating factor, the direct effect (*c′*) remains significant, however with a slightly lower effect (effect 0.59, *p *< 0.05). The indirect effect was not significantly altered (Table 4).

### 3.7 No Aberrant Immune Activation

**FIGURE 5:**
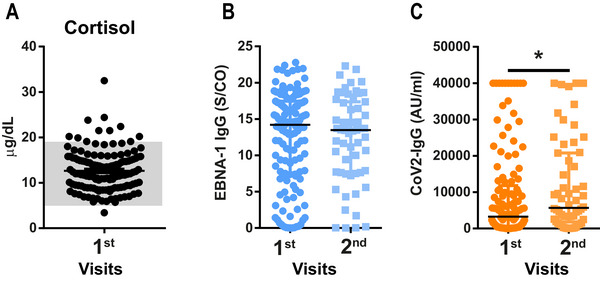
Immune reaction in post‐COVID‐19 condition (PCC) patients: Cortisol levels are within the physiological range between 4.8 and 19.5 µg/dL (*n *= 134; gray area; data shown as median ± IQR). (A) EBNA‐1 IgG titer measurements indicated no change of antibody concentration over time (data shown as median ± IQR; Mann–Whitney test *p *= 0.6). (B) SARS‐CoV‐2 IgG titer measurements indicated an increase in antibody production over time (Mann–Whitney test, *p *= 0.046) (for antibody titer measurements, first visit *n *= 137, second visit *n *= 59) (C).

Median cortisol level was 12 µg/dL (IQR = 9.0–15.3 µg/dL), which is within the physiological range of 4.8–19.5 µg/dL (indicated in gray) with 10 outliers (Figure 5A). To evaluate possible viral reactivation of EBV, we analyzed levels of EBNA‐1 IgG in the serum at the first and second visits. Although most patients were positive for EBNA‐1 IgG, indicating previous EBV contact, antibody titer did not change during the examined time course (Figure 5B). For SARS‐CoV‐2 IgG, we did find a significant increase of IgG from first to second visit (Figure 5C).

## 4 Discussion

Four years after the first description of SARS‐CoV‐2, post‐infectious sequelae are still unclear, and some patients have suffered for years from PCC (Wahlgren et al. 2023; Kim et al. 2023; Xu, Xie, and Al‐Aly 2022). In this study, the initial symptoms became less severe or disappeared completely in most patients over the observation period. However, fatigue and cognitive difficulties influenced daily living longitudinally in patients with persisting PCC. We could not verify previously postulated biomarkers in PCC but found somatization as a main trigger factor for the persistence of multiple symptoms.

Although patients displayed a high symptom load at the first visit, more than half were symptom‐free, and 76.7% had improved clinically at the last follow‐up. Two other studies investigated self‐reported symptoms for 2 years, where one found more than 80% (Wahlgren et al. 2023) still affected, whereas the other one found only a third still suffering from neuropsychiatric symptoms (Kim et al. 2023), pointing toward the high variability in perception of self‐reported symptoms. Furthermore, high drop‐out rates increase the variability of self‐reported recovery rates even more.

To quantify the symptom load for some core symptoms, we performed a battery of well‐established questionnaires and evaluated them longitudinally. We could show that severity of symptoms decreased in fatigue and insomnia scores, whereas daytime sleepiness remained unchanged. Moreover, we were not able to objectivize self‐reported cognitive impairment with the MoCA test. Previous studies reported cognitive impairment based on standardized tests (Taquet et al. 2022; Crivelli et al. 2022; Becker et al. 2021), whereas others did not or only in specific areas, such as attention or executive function, indicating high variability as to the extent of cognitive deficits and the affected domains in PCC, possibly also influenced by sleepiness or insomnia (Ludwig et al. 2023; Aziz et al. 2023; Martin et al. 2023; Petersen et al. 2022).

Overall, 70% of patients previously employed or studying were not able to return to work in full capacity, similar to other study cohorts (Nittas et al. 2022; Kisiel et al. 2022; Kerksieck et al. 2023), and this underlines the significance of PCC‐associated costs that may incur for social systems (Gandjour 2023). Additionally, the high percentage of patients suffering from psychiatric symptoms impedes full recovery and occupational reintegration (Kerksieck et al. 2023).

Patients with a psychiatric diagnosis are highly overrepresented in comparison to the average population in PCC studies (Ludwig et al. 2023; Fleischer et al. 2022; Taquet et al. 2022). In our cohort, 18% were newly diagnosed with a psychiatric disease after the SARS‐CoV‐2 infection. Patients could have suffered from a psychiatric disease before but only sought medical attention after the infection. Furthermore, the pandemic itself is known to cause or aggravate psychiatric symptoms due to social isolation and general insecurity (Salari et al. 2020). Additionally, suffering from PCC symptoms highly affects the quality of life and may be associated with depression and anxiety (Nittas et al. 2022; Rass et al. 2023). Therefore, psychiatric diseases are most likely not directly caused by the virus but are rather the result of various socio‐economic stressors (Nittas et al. 2022; Salari et al. 2020).

In only 3%, an alternative diagnosis was made explaining the patient's symptoms. As patients in our clinic had undergone a broad battery of assessments from other physicians prior to coming to our PCC clinic, many patients with an underlying somatic cause of their symptoms had already been diagnosed as such and were not further referred to our specialized clinic. However, our data indicate that somatization plays a crucial role in the development and persistence of PCC symptoms, with over 60% of patients being affected. Patients with a medical history of psychiatric diseases, but also media coverage of pandemics and the nocebo effect, have been shown to induce somatic symptoms without a physiological correlate (Joffe and Elliott 2023). Moreover, stress, depression, and anxiety have been shown to increase inflammatory markers in the blood, irrespective of previous infections. Therefore, there is not necessarily a clear separation between psychological and physiological causes of symptoms attributed to PCC (Joffe and Elliott 2023). Interestingly, not psychiatric diseases or age but gender was found to be a modulating factor in this study for the number of symptoms developed in consideration of the somatization status. However, this could be a statistical bias due to the high overrepresentation of women in this study.

This confirms recently published data (Kachaner et al. 2022; Horn et al. 2023; Fleischer et al. 2022) and highlights issues future clinical trials have to address when selecting patients for PCC studies. The high levels of media‐induced anxiety, lack of clear diagnostic criteria, and inhomogeneous cohorts impede the selection of a patient cohort for the implementation of well‐designed clinical research (Altmann et al. 2023; Høeg, Ladhani, and Prasad 2023; Srikanth et al. 2023), and urge for a robust biomarker. We investigated cortisol levels in the serum, which have been shown to be decreased in PCC patients (Klein et al. 2023; Su et al. 2022). However, we were not able to replicate those results, as in our cohort most patients were well within the physiological range.

Another prominent hypothesis of the PCC pathomechanism and potential biomarker is reactivation of EBV (Gold et al. 2021; Klein et al. 2023; Su et al. 2022; Peluso et al. 2023). Initial infection with SARS‐CoV‐2 and, hence potential EBV reactivation, was several months past; therefore, we did not test for early markers of EBV reactivation but rather for a long‐term immune response (Peluso et al. 2023; Nystad and Myrmel 2007; Hess 2004). We longitudinally analyzed the EBNA‐1 IgG titer as a marker for previous EBV infection and immune response against it (Peluso et al. 2023; Hess 2004). Most patients were positive for EBNA‐1 IgG, indicating previous exposure to the virus at some point in their life (Nystad and Myrmel 2007; Hess 2004). However, we found stable EBNA‐1 IgG levels over time, which does not support the hypothesis of virus reactivation (Peluso et al. 2023). Hence, we were not able to reproduce the results from previously published studies pointing toward an EBV reactivation (Gold et al. 2021; Klein et al. 2023; Su et al. 2022; Peluso et al. 2023). However, we were in line with another study, where serum levels of blood donors before and after the pandemic were investigated for EBV reactivation. In this study, no increased levels of viral reactivation were found, neither in PCC patients nor in healthy controls (Hoeggerl et al. 2023).

On the other hand, we did find an increase of SARS‐CoV‐2 IgG. This is most likely due to repeated exposure to the virus and the kinetics of antibody development against SARS‐CoV‐2 (Movsisyan et al. 2022). Other studies show low SARS‐CoV‐2 titer but higher response to other viruses, which was interpreted as undirected chronic immune activation (Spatola et al. 2023; Su et al. 2022; Gaebler et al. 2021). However, in this study, we could show that PCC patients do have a high immune response, specifically against SARS‐CoV‐2. This is also in line with a study, which showed good viral clearing in PCC patients over time, indicating a good immune response against SARS‐CoV‐2 (Hoeggerl et al. 2023). Hence, our results do not point toward undirected immune activation but rather to a potent defense against SARS‐CoV‐2, confirming other previously published data (Klein et al. 2023; Hoeggerl et al. 2023).

Or data suggest that long COVID is related to functional neurological disorders. The connection between a Covid infection and functional neurological symptoms is controversially discussed. The main challenge in diagnosing symptoms as functional rests in the low yield of positive features. The diagnosis is therefore mainly made on the basis of missing pathological findings in clinical and paraclinical examinations. However, positive features do exist in functional neurological disorders, most of all temporal inconsistency of symptoms (Teodoro et al. 2023). This was present in the majority of our patients, who reported significant fluctuations in the severity of their complaints, inconsistent with any organic disorder.

There are several ways in which a Covid infection might be related to a functional neurological disorder. Depression is associated with cognitive deficits. The prevalence of depressive disorders has risen during the pandemic (Casjens et al. 2024). Hence, some patients may have suffered from depression. This is of particular relevance due to the high frequency of cognitive symptoms (“brain fog”) in PCC (Ball et al. 2020). Furthermore, media coverage may have induced enhanced introspection. This may trigger other pathophysiological mechanisms associated with functional disorders, such as a disruption in predictive processing, where an imbalance in the integration of sensory data and top‐down predictions leads to the symptoms experienced by the patient (Mavroudis et al. 2024).

There are some strengths and weaknesses in this study that need to be addressed. The study has a prospective design, and patients were examined longitudinally using a comprehensive test battery. Our study lacks a healthy (i.e., people post‐Covid infection who never developed PCC) control group. This impedes data interpretation. The high drop‐out rate in the longitudinal course of this study is one of the major limitations and introduces a potential bias into the study. Study outcomes may have been influenced in two ways. Symptom persistence may be overestimated if drop‐outs were due to sufficient symptom relief in a large proportion of patients. On the other hand, it may have been underestimated if drop‐out indicated discontent or disillusion of more severely affected patients with our clinic. Additional bias may have been introduced by the low completion rate of the questionnaires. Again, this may have been due to some patients experiencing too much fatigue to fill out the questionnaires, whereas others experienced a lack of time due to the resumption of their regular pre‐Covid activities. In practice, we experienced both scenarios.

Furthermore, selection is biased toward a patient cohort with a high complaint load willing to seek medical treatment. However, this cohort is of interest as even though the symptom load is high, an improvement over time was observed. Furthermore, patients self‐administered a broad variety of medical treatments, which could interfere with our analyses.

In summary, we found a high subjective symptom load in patients presenting at the PCC outcome clinic but could not objectify cognitive deficits or laboratory anomalies. Most patients showed significant improvement over time. Notably, 61% of patients were positive for somatization according to the PHQ‐D, indicating a substantial overlap with other PCC symptoms. These findings support pandemic‐related stress and anxiety as important factors contributing to PCC.

This study shows the difficulties in finding objective parameters and biomarkers for PCC, which are the prerequisite for well‐designed clinical trials. The high rate of somatization supports a role for neurologists in the diagnosis of exclusion of organic disease but points out the need for a multidisciplinary team, including psychosomatic support, to properly manage this disease.

## Author Contributions

**Anna Tröscher**: conceptualization, data curation, formal analysis, methodology, project administration, visualization. **Patrick Gebetsroither**: data curation, investigation, methodology. **Marc Rindler**: data curation. **Vincent Böhm**: data curation, writing–review and editing. **Rainer Dormann**: investigation. **Tim von Oertzen**: conceptualization. **Anna Heidbreder**: writing–review and editing. **Raimund Helbok**: writing–review and editing, supervision. **Judith Wagner**: supervision, conceptualization, methodology, project administration, writing–review and editing.

## Consent

Consent was obtained from every patient.

## Conflicts of Interest

Judith Wagner received speaker fees from Janssen‐Cilag GmbH, UCB Pharma AG, Boehringer Ingelheim, congress/travel funds from Novartis Pharma GmbH, Roche Austria GmbH, Boehringer Ingelheim, and added board reimbursements from Janssen‐Cilag GmbH and Novartis Pharma GmbH. Vincent Böhm received travel funds from Novartis Pharma GmbH. Tim von Oertzen received grants from FFG and Böhringer Ingelheim, consulting fees or honoraria for lectures from Angelini Pharma, Arvelle, GW Pharma, Jazz Pharma, Biogen, LivaNova plc and Eisai, travel and meeting support from Angelini Pharma and Jazz Pharma. All other authors report no conflict of interest.

### Peer Review

The peer review history for this article is available at https://publons.com/publon/10.1002/brb3.70087.

## Supporting information



Table S1: Pathological Blood Parameter findings and their frequency.

## Data Availability

The data that support the findings of this study are available from the corresponding author upon reasonable request.
