# Supplementary material for: High Somatization Rates, Frequent Spontaneous Recovery, and a Lack of Organic Biomarkers in Post‐Covid‐19 Condition
Source: Brain Behav. 2024 Oct 8;14(10):e70087. doi: 10.1002/brb3.70087 (PMC11460636; doi:10.1002/brb3.70087)
Supplement: Supplementary file 1 — Table S1: Pathological Blood Parameter findings and their frequency. [file BRB3-14-e70087-s001.docx]

Supplementary Table 1: Pathological Blood Parameter findings and their frequency

| **Parameter** | **Percentage affected** |
| --- | --- |
| Elevated creatine kinase | 9/127 (7 %) |
| Low iron metabolism markers | 14/127 (11 %) |
| High Vitamin B12 | 6/127 (5 %) |
| High glucose levels | 3/127 (2 %) |
| Other pathological blood parameters (e.g. high alanine aminotransferase, high coagulation time  or high sodium) | 12/127 (9 %) |
